# A Genetic and Immunohistochemical Analysis of *Helicobacter pylori* Phenotypes and *p27* Expression in Adenocarcinoma Patients in Jordan

**DOI:** 10.1007/s44197-023-00099-z

**Published:** 2023-04-18

**Authors:** Suhaila A. Al-Sheboul, Ahmad Abdul-Razzak Mohammad, Yasemin Shboul, Brent Brown, Ismail I. Matalka

**Affiliations:** 1 Department of Medical Laboratory Sciences, Faculty of Applied Medical Sciences, Jordan University of Science and Technology (JUST), Irbid, 22110 Jordan; 2 Biochem123Education, London, UK; 3 Department of Pathology and Microbiology, Faculty of Medicine, Jordan University of Science and Technology (JUST), Irbid, 22110 Jordan

**Keywords:** Formalin-fixed paraffin-embedded tissue blocks, Gastric cancer, Gastrectomy, *HelicobacterpylorivacA-cagA*, Immunohistochemistry, p27

## Abstract

Stomach (gastric) cancer is one of the most prevalent and deadly cancers worldwide and most gastric cancers are adenocarcinomas. Based on prior research, there is an association between *Helicobacter pylori* (*H. pylori*) infection together with the frequency of duodenal ulcer, distal gastric adenocarcinoma, mucosa-associated lymphoid tissue (MALT) lymphoma, and antral gastritis. *Helicobacter pylori* virulence and toxicity factors have been identified before that significantly influence the clinical outcomes of *H. pylori* infection and gastric adenocarcinoma. However, it remains unclear exactly how different strains of *H. pylori* affect gastric adenocarcinoma. Current research suggests this involves tumor suppressor genes, like *p27* but also *H. pylori* toxic virulence proteins. Therefore, we quantified known *H. pylori* genotypes within adenocarcinoma patients to establish the prevalence of known toxins that include cytotoxin-associated gene A (c*agA*) as well as vacuolating cytotoxin A (v*acA*) within patients of variable adenocarcinoma diagnosis. This analysis used gastrectomy samples validated for DNA viability. The incidence of *H. pylori* in adenocarcinoma patients in Jordan was established to be 54.5% positive (*ureA* gene positive) with c*agA* genotype occurrence at 57.1%, but also in this population study v*acA* gene ratios found to be 24.7%:22.1%:14.3%:14.3%. (*vacAs1:vacAs2:vacAm1:vacAm2*). Using immunohistochemistry (IHC), we confirmed with statistical significance that p27 was dysregulated and suppressed, within nearly all *H. pylori vacA* genotypes. In addition, within 24.6% of *H. pylori* samples analyzed was a different bacterial genotype, and curiously that p27 protein expression was retained in 12% of tested adenocarcinoma *H. pylori* samples. This is suggestive that p27 could be used as a prognostic indicator but also that an unknown genotype could be contributing to the regulatory effects of p27 protein within this bacterial and cellular environment that may include other virulence factors and unknown immune system regulatory changes.

**Supplementary Information:**

The online version contains supplementary material available at 10.1007/s44197-023-00099-z.

## Introduction

### Background

**Fig. 1:**
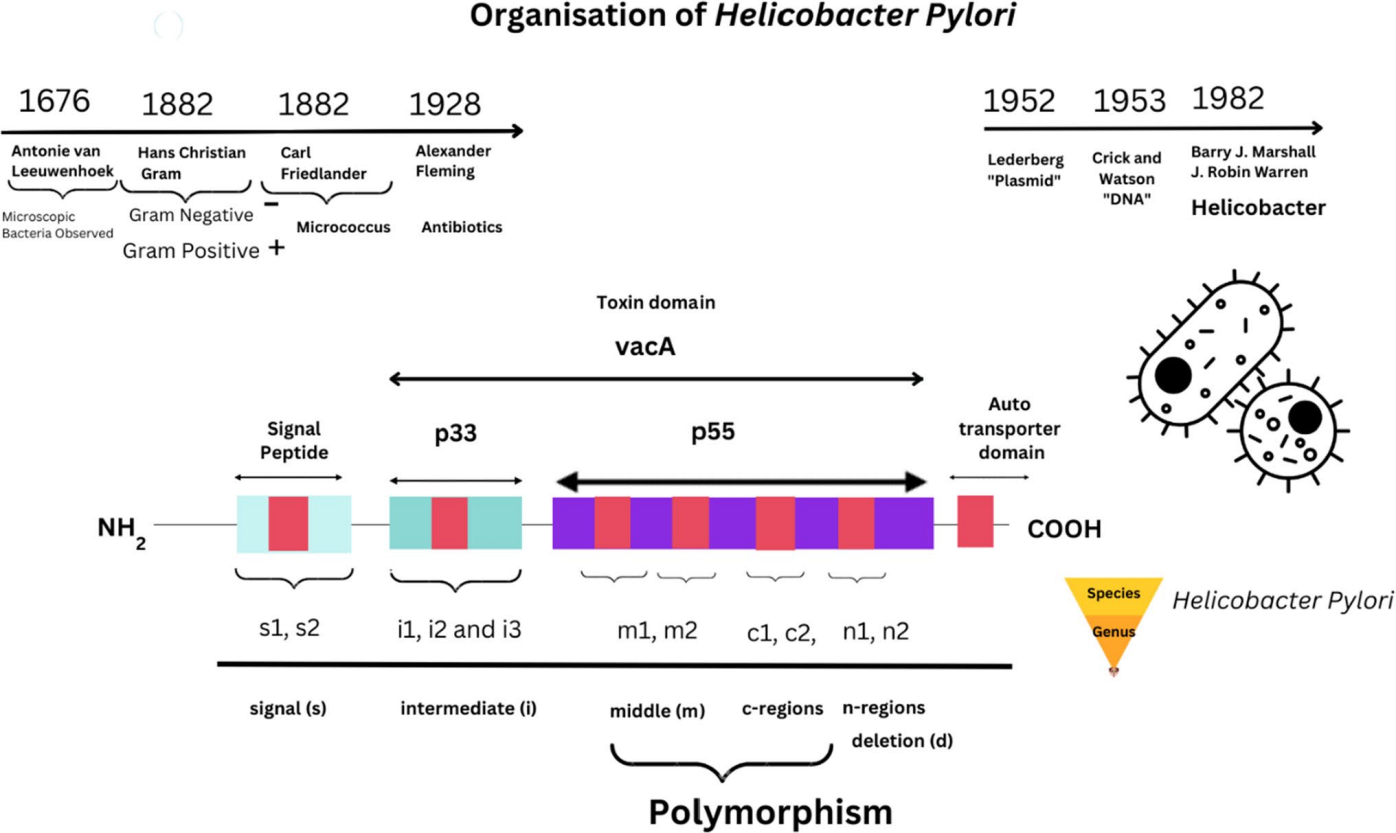
Perspectives of *Helicobacter pylori* genome

Stomach cancer (also known as gastric cancer) is the third most common cancer-related cause of death worldwide and 95% are adenocarcinomas [1]. Adenocarcinomas develop from glands of the stomach mucosa, or its most superficial layer [1]. However, there are additional cancers that can develop from the stomach, such as leiomyosarcomas, which develop from the muscles that surround the mucosa, and mucosal associated lymphoid tissues (MALT) lymphomas [1]. The MALT is composed of immune system cells that include B and T cells, but also those that present both pathogenic as well as tumor associated antigens (TAA). These include monocytes, macrophages and dendritic cells (DCs) that modulate the systemic immune response before, during and after disease. Cytokines (interleukins or IL) can be expressed and secreted within epithelial cell layers surrounding gastric tissues that include IL–8, tumor necrosis factor (TNF–α) and interferons (type I/II/III). Gastric cancers are frequently discovered at advanced stages, so the prognosis can be poor [2]. *Helicobacter pylori* was classified as a class 1 carcinogen by the World Health Organisation (WHO) in 1994, with epidemiological, clinical, and experimental data demonstrating a link between *H. pylori* infection and the progression of gastric adenocarcinoma [2]. *Helicobacter pylori* is considered to thrive in the acidic pH environment of the stomach. Prior reports suggest that three classifications existed for adenocarcinoma with a 2018 report defining five classifications. These include papillary, tubular, poorly cohesive, mucinous, weakly coherent, with various histological variants (e.g., squamous cell carcinoma) (see Supplementary Materials). Individuals with *H. pylori* infection are indicated to have a 6–fold higher chance of developing gastric cancer [3]. Prior reports suggest that greater than 50% of the global population are infected by *H. pylori* [4] The presence of this bacterium increases the risk of developing gastric adenocarcinoma [5]. *Helicobacter pylori* possess virulence factor genes that affect toxicity and pathogenicity including cytotoxin–associated pathogenicity island (*cagPAI*) and vacuolating cytotoxin A (*vacA*) through proteins affecting *H. pylori* virulence (see Fig. 1).

These encode proteins that include vacA toxin (molecular mass 140 kDa), initially formed from a protoxin. VacA was originally named after its ability to form vacuolar like membrane vesicles within gastric epithelial cells that enhance *H. pylori* colonization of the gastrointestinal (GI) tract. In addition, within v*acA* genes, approximately 30 genes encode *cagPAI* (molecular mass 40 kDa) proteins that affect components of the intestinal type IV secretion system (T4SSs) protein. These are present in other Gram negative (-ve) bacteria [6–9]. The T4SS proteins assemble through protein pili–like structures encoded by various other genes that code for other cagA proteins (e.g., CagI, CagY etc.), whilst delivering cagA protein into host cells upon bacterial attachment utilizing adhesion molecules [8, 10]. Other types of secretion systems have been elucidated as there are 6 types known [6–9]. Moreover, currently data on Uniprot indicates that *cagPAI* genes encode 378 proteins categorized now (see Supplementary Materials). These toxic virulence factors have been proven to be crucial in defining clinical outcomes of *H. pylori* infection and development of gastric adenocarcinoma [10]. Prior laboratory studies have demonstrated the *cagA* gene role in carcinogenesis through stimulation of aberrant cell proliferation [11]. Over 90% of *H. pylori* strains occur in Southeast Asia occur with more than 60% of strains from Europe and North America that are reported to possess the *cagA* gene [12]. In general, vacA strains encompass type s1a and type m1 that produce greater toxin amounts, followed by type s1b and type m2 strains, which generate toxin in moderate amounts, while vacA type s2 and type m2 strains exhibit less or no vacuolar toxin activity [13, 14]. The presence of various genotypes of *vacA* has been reported, including s1a, s1b, s2, ml, and m2 strains of *H. pylori* that display unusually variable toxicity [15]. Therefore, characterizing the toxicity of *H. pylori* in specific carcinomas allows further knowledge of virulence factors within clinically diverse and vulnerable patients.

### Background and Molecular Mechanisms of* Helicobacter pylori*

Vacuolating cytotoxin A gene is considered to have diverse polymorphisms in allelic expression within the signal (s), middle (m), intermediate (i), deletion (d) and c regions [16, 17]. Current data on Uniprot are indicative of 3794 variations within *H. pylori vacA* genes (see Supplementary Materials). The *vacA* gene produces a toxin composed of two subunits (p33/p55) considered to target cellular mitochondria [4, 18]. These are known to bind to other cellular integrin receptors. VacA is suggested to enter phospholipid cell layers by forming a membrane pore, and then as vesicle like endosomes. Past research indicates that anion selective channels are created, with the p55 subunit required for membrane binding. As a result, increasing transport of chloride ions to change the mitochondrial inner membrane electrochemical potential [4, 18]. This homeostatic balance within the GI tract can therefore, affect the immune system with differential effects on not only regulatory T cells, but also helper T cell, cytotoxic T cell, and antigen presenting cells (APCs), as well as B cells together with natural killer cells. These are essential in immune systems of clinically diagnosed cancer patients. Either therapeutic or cancer related immunosuppression can therefore contribute to prolonged microbial infection thereby affecting disease outcomes [19]. *Helicobacter pylori* also secrete a urease enzyme (550kDa) that catalyzes hydrolysis into products of ammonia and carbonic acid. Urease is encoded by a fused *ure* gene cluster (*ureA* and *ureB*) that encodes other accessory proteins (UreE, UreF, UreI, etc) [20]. Urease is thought to elicit a strong B cell serum immunoglobulin response and facilitate host colonization [20]. Therefore, in combination with VacA toxin these contribute to prolonged infection in the GI tract. Concurrent initiation occurs by upregulation of nuclear transcription factor (NF–kB) in combination with upregulation of the cytokine interleukin–8 (IL–8) that are both crucial factors [21, 22] Recently it has also been suggested that IL-8 could also act as a chemoattractant (named CXCR8) for Epstein Barr Virus (EBV) infected B cells [21]. The p27 protein (often referred to as KIP1) is a member of the cyclin-dependent kinase inhibitor (CDKI) KIP family of tumor-suppressor proteins regulated by transforming growth factor beta (TGF–β) [20]. One proposed mechanism for the development of cancer is the inactivation of this p27^KIP1^ tumor suppressor gene, located on chromosome 12p13 through transcriptional regulation [23, 24]. Eukaryotic cells have a network of regulatory proteins that affect cell cycle control, which regulate and control the cell cycle to prevent malignant cell proliferation and cancer [23]. Activities of the cell cycle checkpoints are governed by cyclin-dependent kinases (CDKs), a family of protein kinases that bind to regulatory proteins known as cyclins [23, 25]. Inhibition of CDKs (e.g., CDK2) is one mechanism that p27 uses to inhibit cell cycle progression, although cell division, proliferation, and apoptosis are other roles of p27 [26]. Reduced p27 expression has been shown to be a marker of aggressive cancer and poor prognosis, including colon, breast, malignant melanoma, liver, stomach, lung, as well as brain tumors [27]. Furthermore, *H. pylori* infection in gastric cancer patients has been associated with reduced p27 expression [23, 28]. C–terminal phosphorylation regulates p27 function through known growth and survival factor phosphoinositide 3–kinases (PI3K) and protein kinase B (AKT) that regulate growth (G1) to synthesis (S) cell cycle progression. *Helicobacter pylori* and CagA protein may also bind to other cytosolic proteins like Csk, Src homology 2 domain-containing tyrosine phosphatase–2 (Shp–2) or c–jun. VacA is considered to bind to and enter gastric cells, as a pore forming toxin, utilizing receptors that include the epidermal growth factor (EGR) receptor, but also heparin sulphate amongst others. [4]. Within immune cell compartments as we discussed in our last paper cellular markers can clarify individual cell types [29]. Recent research is evocative that VacA toxin can utilize a cluster of differentiation molecules (CD18) β2 integrin adhesion subunit molecule expressed on T cells that forms part of the CD11a/CD18 transmembrane receptor leukocyte function associated antigen (LFA–1) complex as well as an enzyme like γ-glutamyl transferase (GGT) central to amino acid transfer and leukotriene synthesis [19, 30]. Little is known about the immunology of *H. pylori* infection; however, it is indicated that the cytokines, IFN–γ and TNF–α, play a protective role [31]. One study investigating *H. pylori in vivo* so far has suggested that cagA protein can suppress DC function [32]. However early research studies suggest that cagA can translocate into APCs (monocytes, macrophages and dendritic cells) [33]. More recently 889 disease enhanced genes (DEGs) were investigated *in vitro* during *Helicobacter pylori* infection [33]. In this study it was suggested that Toll-like receptor 4 (TLR4) together with chemokine CXCR3 expression could be modulated by *H. pylori* infection specifically by cagA protein [33].

### *Helicobacter pylori* Epidemiology and Other Factors

In several prior studies conducted around the world, *cagA*, *vacAs1*, and *vacAm1* genes, are considered to play a role in pathogenicity and linked to gastric cancer [14, 36, 37]. The geographic distribution of *H. pylori* strains varies. For instance, the most common strains in East Asia carry the *cagA*, *vacAs1*, and *vacAm1* genes [38]. The prevalence of *vacAs1b* subtype was approximately 100% in South America, 80% in the Spain and Portugal strains, and low in East Asia [38]. The mechanism of the development of gastric adenocarcinoma in *H. pylori*-infected samples has not yet been fully determined [39]. Establishing causal genetic factors therefore may eventually clarify relationship with antimicrobial resistance. Some earlier reports between 2000 and 2005 quantify changes in antimicrobial resistance within *H. pylori* infection to metronidazole, clarithromycin, amoxicillin and tetracycline [40]. Other reviews consider antimicrobial resistance further in 2023 during *H. pylori* infection [41].

In this investigation, we therefore sought to identify the relationship between *H. pylori* infection and p27 expression in gastric cancer tissues in Jordanian patients. We utilized gastrectomy samples rather than tissue biopsies taken from patients with *H. pylori.* These samples were clinically diagnosed with variable adenocarcinoma grades from hospitals in Jordan. *Helicobacter pylori* genotypes of each clinical sample were characterized alongside p27 protein expression confirmation. To ascertain whether p27 protein was related to the development of gastric cancer in these patients, the level of p27 gene expression was also assessed. To our knowledge, this is the only study that has quantified variable *H. pylori* genotypes in adenocarcinoma with confirmation of expression of p27 protein using FFPE-gastrectomy samples obtained from patients in Jordan.

## Materials and Methods

### Sample Collection

In this study, archived histological samples (n=77) with linked patient demographic and clinical data were obtained within 8 years (2005 and 2013) from the pathology department medical records of King Abdullah University Hospital (KAUH) at Jordan University of Science and Technology (JUST) (Irbid, Jordan) and the Jordanian Royal Medical Services (JRMS) (Amman, Jordan) (Table 1). These clinical samples (*n* = 77) were examined using formalin-fixed paraffin-embedded tissue analysis (FFPE). All samples were classified as gastric adenocarcinomas and stored at 25 ℃. This study was carried out with consent from the Institutional Review Board (IRB), Ethics Committee, at Jordan University of Science and Technology (Ref: 20/51/201).

**Table 1:** PCR protocol for *H. pylori* virulence gene amplification

*Helicobacter pylori* gene	Cycling profile × 40
*cagA*	95 °C for 10 min 95 °C for 1 min 61 °C for 1 min 72 °C for 1 min 72 ℃ for 10 min
*vacAs1*	95 °C for 10 min 95 °C for 1 min 54 °C for 1 min 72 °C for 1 min 72 ℃ for 10 min
*vacAs2*	95 ℃ for 10 min 95 ℃ for 1 min 52 ℃ for 1 min 72 ℃ for 1 min 72 ℃ for 10 min
*vacAm1*	95 ℃ for 10 min 95 ℃ for 1 min 48 ℃ for 1 min 72 ℃ for 1 min 72 ℃ for 10 min
*vacAm2*	95 ℃ for 10 min 95 ℃ for 1 min 52 ℃ for 1 min 72 ℃ for 1 min 72 ℃ for 10 min

### Clinical Samples Processing

Using a microtome, formalin-fixed tumor specimens were embedded in paraffin blocks and sectioned into 5 µm thick tissue slices (Energy beam science, East Granby, CT, USA). The microtome blade was changed and cutting surface both cleaned and sterilized with xylene and absolute ethanol (100%), respectively. Up to 10 tissue sections were collected in 1.5 ml sterile Eppendorf nuclease free tubes for DNA extraction, and 5 µm tissue slices were mounted on positively charged slides for IHC.

### Immunohistochemistry (IHC) Staining

To evaluate p27^KIP1^ expression in tissue(s), all tissue section slides were treated with a mouse monoclonal anti-human IgG p27Kip1 antibody (Clone SX53G8.5 dilution 1:100; Code M7203; Dako Cytomation, Denmark) that cross-reacts with p27kip1 using a protocol described previously [42]. Slides were deparaffinized with xylene twice, for 5 minutes each, rehydrated through a series of graded alcohol washes, 2 times, for 3 minutes each, and then transferred once through 95%, 70%, and 50% alcohols for 3 minutes each). Endogenous peroxidase activity was blocked by incubating sections in 3% H_2_O_2_ solution in methanol at room temperature for 10 min. Then slides were rinsed with PBS twice, for 5 min each. To reveal the antigenic epitope, we performed antigen retrieval by pouring 300 mL of 10 mM citrate buffer, pH 6.0 into the staining container containing arranged slides, and incubating at 95 °C for 23 minutes using a pretreatment (PT) system (Dako, Agilent, Glostrup, Denmark). We removed the staining container at room temperature and allowed the slides to cool for 20 min. This was followed by washing in Dulbecco’s phosphate-buffered saline (PBS) twice, for 5 min each (Sigma Aldrich, St. Louis, MO, USA). Then, blocking buffer was drained from the slides. All slides were treated with p27 monoclonal antibody (H-1): sx-53G8.5 (Dako Cytomation, Agilent), diluted 1:100 in Dako antibody diluent, for 45 min at room temperature. Slides were washed with PBS twice, for 5 min each, according to manufacturer recommendation [42]. The detection was carried out using Dako EnVision®+ Dual Link System-HRP (DAB+) (Dako, Agilent). The Liquid DAB+ Substrate Chromogen System (Dako, Agilent) was used to view the slides, and Mayer’s hematoxylin (Polysciences) was used as a counterstain. As a negative control, primary antibodies were excluded. Tonsil and lymphoma patient tissue was used as a positive control. More than 5% of neoplastic cells should exhibit prominent brown nuclear staining for samples to be deemed positive for p27 protein expression. The scoring system used to evaluate the results of immunostaining was described previously [43]. To verify the initial diagnosis, all samples were examined and confirmed by a licensed pathologist at the pathology division of KAUH. All cells were counted on the slides using a high-power field scanner, and pictures were taken with a digital camera (Olympus model C-5060, Olympus, Tokyo, Japan).

### DNA Extraction

For extracting genomic DNA, 5–10 sections from each block were placed into sterile, nuclease-free Eppendorf tubes. Prior to genomic DNA extraction, xylene and paraffin residues from paraffin sections were removed in the pathology lab in accordance with standard operating procedures [44]. To quickly and effectively deparaffinize the tissue, 1 mL of xylene was added to each tube, with tubes vortexed violently for 10 s. The mixture was centrifuged at a full speed of 4000 rpm for 2 min at room temperature, with pellet remaining in the tube. This procedure was performed twice. One mL of 96% ethanol was added to each pellet, vortexed and centrifuged at full speed for 2 min at room temperature, and supernatant removed by pipetting. To remove any leftover xylene, this step was repeated. Next, Eppendorf tubes containing the tissue pellets were opened and incubated at 37 °C for 15–30 min until the ethanol evaporated. Each pellet was then subjected to genomic DNA extraction using a QIAamp DNA FFPE Tissue kit (Qiagen, Hilden, Germany) according to manufacturer instructions. To ensure DNA purity, the extracted DNA was eluted, and concentration assessed using a NanoDrop ND-1000 Spectrophotometer (Thermo Scientific, Waltham, MA, USA). For later use, all DNA extracts were kept in storage at −20 °C. Using an antibody to Ki-67, adjacent sections were immune-stained to detect proliferating cells, and apoptotic cells were detected using a terminal deoxynucleotidyl transferase-mediated dUTP-biotin nick end labeling assay. Proliferation and apoptotic indices were calculated as previously described [42].

### Detection of *H*. *pylori* Virulence Genes

*Helicobacter pylori* virulence genes were detected by PCR amplification. The primers used here are described below (see Tables 1 and 2). Primers targeted amplification of *H. pylori* genes and the glyceraldehyde 3-phosphate dehydrogenase (GAPDH) gene as an indicator of DNA extraction validity and viability [45].

**Table 2:** PCR primers for *H. pylori* genes (and GADPH quality control bottom)

Gene	Primer	Sequence	Size *	Ref.
*ureA*	F 'R	5'-GCC AAT GGT AAA TTA GTT-3' 5'-CTC CTT AAT TGT TTT TAC-3'	411	[46]
*cagA*	F & R	5’-AAT ACA CCA ACG CCT CCA AG-3’ 5’-TTG TTG CCG CTT TTG CTC TC-3’	400	[47]
*vacA*s1	F & R	5’-ATG GAA ATA CAA CAA ACA CAC-3’ 5’-CTG CTT GAA TGC GCC AAAC-3’	259	[48]
*vacA*m1	F & R	5’-GGT CAA AAT GCG GTC ATG G-3’ 5’-CCA TTG GTA CCT GTA GAA AC-3	290	[48]
*vacA*m2	F & R	5’-GGA GCC CCA GGA AAC ATT G-3’ 5’-CAT AAC TAG CGC CTT GCA C-3’	352	[48]
*vacA*s2	F & R	5’-ATG GAA ATA CAA CAA ACA CAC-3’ 5’-CTG CTT GAA TGC GCC AAA C-3’	286	[48]
*GAPDH*	F & R	5′-GGC CTC CAA GGA GTA AGA CC-3′ 5′-CCC CTC TTC AAG GGG TCT AC-3′	157	[49]

### PCR for the GAPDH Gene

One of the universal housekeeping genes used to assess the integrity of DNA samples is GAPDH and is used as an internal control. This gene was subjected to PCR. Each PCR reaction with a total volume of 25 µL contained 12.5 µL of PCR master mix (Promega; Madison, WI, USA), 8.0 µL of nuclease free water, 1.0 µL (5 pmol/µL for GAPDH) of each, and 2.5 µL of DNA (100 ng/µL). A negative control reaction (containing all components except DNA template) was included. Amplification protocol was run with the thermal profile recommended by the master mix manufacturer; initial denaturation at 95 °C for 5 min, followed by 40 cycles of denaturation at 95 °C for 1 min, annealing at 54 °C for 1 min, and extension at 72 °C for 1 min. The amplification ended with a final extension at 72 °C for 5 min.

### PCR ureA Gene to Confirm* H. pylori *Identity

PCR for detection of the *H. pylori ureA* gene was performed in 25 µL volumes as described above, using specific *H. pylori ureA* primers along with positive and negative controls. The amplification protocol was run with the thermal profile recommended by the manufacturer; initial denaturation at 95 ℃ for 10 min, then followed by 35 cycles of denaturation at 94 ℃ for 1 min, annealing at 47 ℃ for 1 min, and extension at 72 ℃ for 1 min.

### *PCR *for* H. pylori *Virulence Genes

Samples positive for *ureA* gene underwent five PCR cycles to detect the presence of virulence genes of *H. pylori* (*cagA, vacAs1, vacAs2, vacAm1*, and *vacAm2*). PCR reactions were performed in 25 µL volumes (see Table 2 for temperature profile for each gene PCR cycle. The resulting amplicons and their corresponding lengths were visualized and documented via gel electrophoresis and analysis (Quantity 1 software, Bio-rad, CA, USA).

### Statistical Analysis

All data were analyzed using SPSS version 19.0 software (SPSS, Inc., Chicago, IL, USA). Pearson’s chi-square (χ^2^ or Fisher test was used to analyze the statistical relationship between *H. pylori* infection, virulence genes, as well as *p27* gene expression in the gastric cancer patients analyzed. At *p* ≤ 0.05, the results were considered significant.

## Results

### Demographic and Clinical Data

Patient ages ranged from 30 to 98 years at diagnosis with two groups, females (*n*=35) and males (*n*=42). Results showed that the gastric cancer cases (*n*=77), 39 were differentially diagnosed with diffuse type cancer and 38 were diagnosed with intestinal type (see Table 3 for summary of the demographic and clinical information for the two groups.)

**Table 3:** Gastric sample classification

Sample	Year	Age	Sex	Diagnosis	Type
1	2003	59	Female	Moderately differentiated adenocarcinoma	Diffuse
2	2003	61	Male	Moderate chronic gastritis	Intestinal
3	2003	59	Male	Invasive moderately differentiated adenocarcinoma	Diffuse
4	2004	76	Male	Moderately differentiated adenocarcinoma	Intestinal
5	2004	54	Male	Invasive adenocarcinoma	Diffuse
6	2004	83	Male	Invasive moderately to poorly differentiated adenocarcinoma	Diffuse
7	2004	84	Male	Moderately differentiated adenocarcinoma	Diffuse
8	2004	43	Male	Poorly differentiated adenocarcinoma	Diffuse
9	2005	50	Female	Invasive moderately differentiated adenocarcinoma	Intestinal
10	2005	81	Male	Moderately differentiated adenocarcinoma	Diffuse
11	2005	66	Female	Poorly differentiated adenocarcinoma	Diffuse
12	2005	33	Female	Invasive differentiated adenocarcinoma	Intestinal
13	2005	45	Female	Poorly differentiated invasive adenocarcinoma	Diffuse
14	2005	71	Male	Invasive moderately differentiated adenocarcinoma	Intestinal
15	2005	51	Female	Invasive poorly differentiated adenocarcinoma	Diffuse
16	2005	44	Female	Invasive differentiated adenocarcinoma	Intestinal
17	2006	98	Female	Poorly differentiated adenocarcinoma	Intestinal
18	2006	51	Female	Poorly differentiated adenocarcinoma	Diffuse
19	2006	83	Male	Poorly differentiated Invasive gastric adenocarcinoma	Intestinal
20	2007	54	Male	Poorly differentiated adenocarcinoma	Diffuse
21	2007	69	Female	Poorly differentiated adenocarcinoma	Intestinal
22	2007	79	Male	Poorly differentiated adenocarcinoma	Diffuse
23	2007	52	Female	Poorly differentiated adenocarcinoma with ulcerated surface	Intestinal
24	2007	73	Male	Moderately differentiated adenocarcinoma	Diffuse
25	2007	76	Female	Moderately differentiated adenocarcinoma	Intestinal
26	2007	82	Male	Poorly differentiated adenocarcinoma	Intestinal
27	2007	75	Male	Poorly differentiated invasive adenocarcinoma	Diffuse
28	2007	47	Male	Poorly differentiated adenocarcinoma with large area of necrosis	Diffuse
29	2007	67	Female	Poorly differentiated adenocarcinoma	Diffuse
30	2007	77	Male	Moderately differentiated adenocarcinoma	Intestinal
31	2008	37	Female	Poorly differentiated adenocarcinoma	Diffuse
32	2008	52	Female	Moderately differentiated adenocarcinoma	Intestinal
33	2008	71	Male	Moderately differentiated adenocarcinoma	Intestinal
34	2008	30	Female	Poorly differentiated gastric adenocarcinoma	Diffuse
35	2008	42	Female	Poorly differentiated adenocarcinoma	Diffuse
36	2009	44	Female	Poorly differentiated adenocarcinoma with endocrine differentiation	Diffuse
37	2009	48	Male	Poorly differentiated adenocarcinoma	Diffuse
38	2009	52	Male	Moderately differentiated ulcerated adenocarcinoma	Intestinal
39	2009	58	Female	Poorly differentiated adenocarcinoma	Intestinal
40	2009	75	Male	Moderately differentiated adenocarcinoma	Intestinal
41	2009	46	Male	Moderately differentiated adenocarcinoma	Intestinal
42	2009	77	Female	Poorly differentiated gastric adenocarcinoma	Diffuse
43	2010	80	Female	Poorly differentiated adenocarcinoma reaching subserosa	Intestinal
44	2010	55	Female	Poorly differentiated adenocarcinoma	Intestinal
45	2010	43	Female	Poorly differentiated adenocarcinoma	Diffuse
46	2010	50	Female	Moderately differentiated adenocarcinoma	Intestinal
47	2010	49	Female	Poorly differentiated adenocarcinoma	Diffuse
48	2010	56	Male	Poorly differentiated adenocarcinoma	Intestinal
49	2011	75	Male	Moderately differentiated adenocarcinoma	Intestinal
50	2011	67	Female	Poorly differentiated adenocarcinoma	Diffuse
51	2011	40	Male	Poorly differentiated adenocarcinoma	Intestinal
52	2012	61	Male	Poorly differentiated adenocarcinoma	Intestinal
53	2012	32	Female	Poorly differentiated adenocarcinoma	Diffuse
54	2012	62	Female	Poorly differentiated adenocarcinoma	Diffuse
55	2010	68	Male	Poorly differentiated gastric adenocarcinoma	Diffuse
56	2009	40	Female	Poorly differentiated adenocarcinoma	Diffuse
57	2009	70	Female	Poorly differentiated adenocarcinoma	Diffuse
58	2012	81	Male	Poorly differentiated gastric adenocarcinoma	Diffuse
59	2008	78	Male	Moderately differentiated adenocarcinoma	Intestinal
60	2010	72	Male	Moderately differentiated adenocarcinoma	Intestinal
61	2010	45	Female	Poorly differentiated adenocarcinoma	Diffuse
62	2011	70	Female	Moderately to poorly differentiated adenocarcinoma	Intestinal
63	2012	69	Male	Poorly differentiated adenocarcinoma	Diffuse
64	2012	86	Male	Moderately differentiated adenocarcinoma	Intestinal
65	2011	68	Female	Moderately to poorly differentiated adenocarcinoma	Intestinal
66	2010	70	Male	Moderately differentiated adenocarcinoma	Intestinal
67	2012	66	Male	Poorly differentiated adenocarcinoma	Diffuse
68	2010	52	Male	Moderately differentiated adenocarcinoma	Intestinal
69	2010	65	Female	Poorly differentiated adenocarcinoma	Intestinal
70	2012	63	Male	Moderately differentiated adenocarcinoma	Intestinal
71	2010	51	Male	Poorly differentiated adenocarcinoma	Diffuse
72	2012	58	Male	Poorly differentiated adenocarcinoma	Diffuse
73	2012	83	Male	Poorly differentiated adenocarcinoma	Diffuse
74	2010	60	Male	Poorly differentiated adenocarcinoma	Intestinal
75	2011	50	Female	Poorly differentiated adenocarcinoma	Diffuse
76	2013	75	Male	Moderately differentiated adenocarcinoma	Intestinal
77	2012	54	Male	Poorly differentiated adenocarcinoma	Diffuse

### Immunohistochemistry Results

**Fig. 2:**
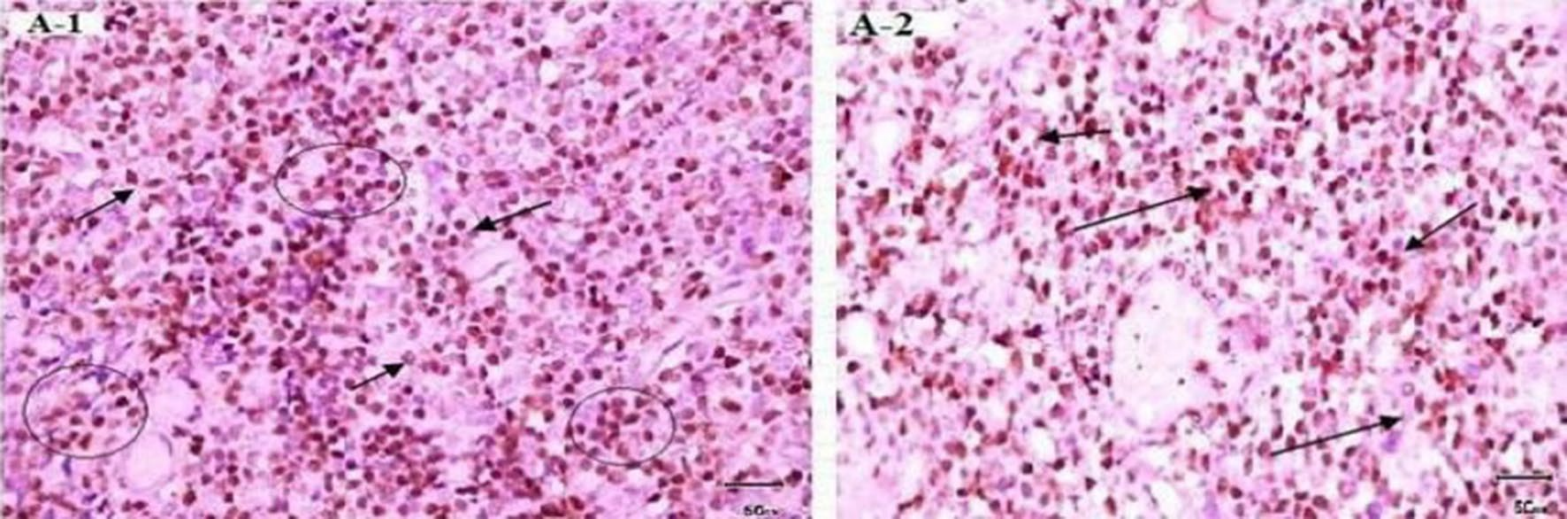
Immunohistochemistry staining of positive controls for p27. A-1: Positive control from tonsil tissue section showing cell staining (brown) of tonsil tissue sample (×10). A-2: Positive control from lymphoma tissue showing cell nuclei staining (brown) in lymphoma sample (×40). Primary antibody anti-p27, secondary antibody anti-Ki67 with peroxidase, DAB chromogen and hematoxylin stain

**Fig. 3:**
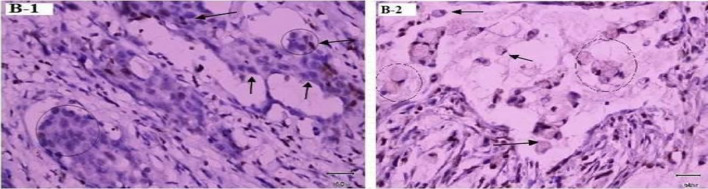
Immunohistochemistry of negative control for P27 expression in cancer tissue. B-1: Negative control from *H. pylori* uninfected gastric intestinal adenocarcinoma sample showing nuclei staining only. Low power (×10) view. B-2: Negative control from *H. pylori* un-infected gastric diffuse adenocarcinoma showing blue nuclear stain around gastric diffuse adenocarcinoma sample. Low power (×10) view. No primary antibody, secondary antibody, anti-Ki67 with peroxidase, DAB chromogen and hematoxylin stain

**Fig. 4:**
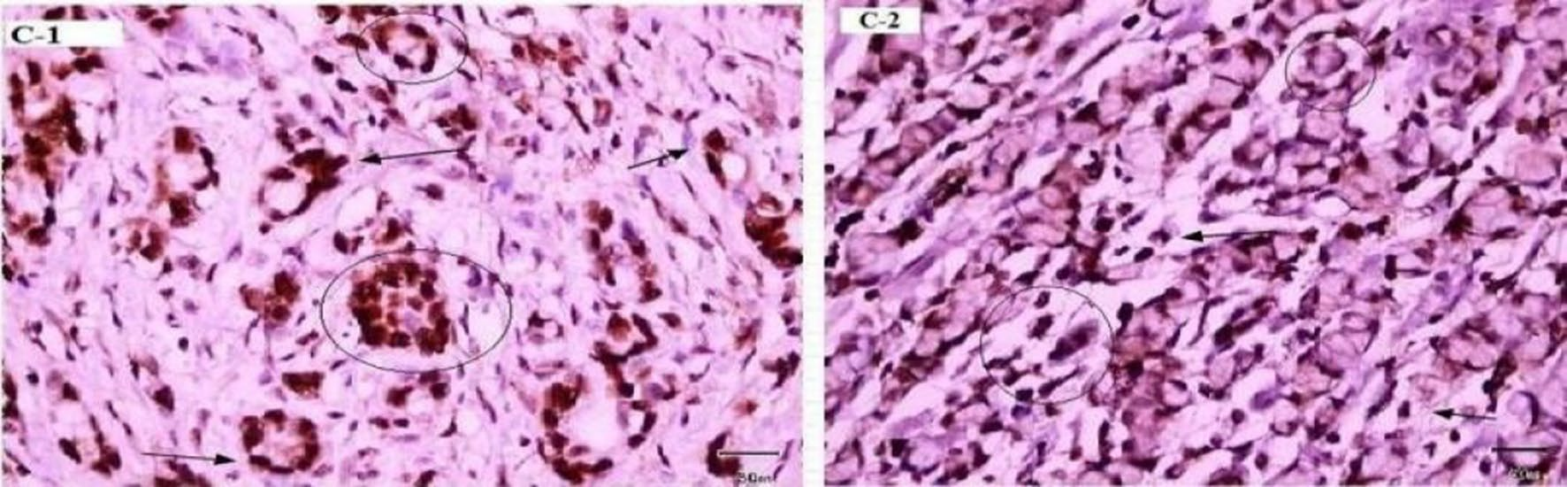
Immunohistochemical Expression of p27 and Ki67 in Cancer Cells. C-1: Immunohistochemical detection of p27 and Ki67 expression. Low power (×10) view of clear positive (brown stain) in epithelium of *H. pylori* infected gastric intestinal adenocarcinoma sample. C-2: Immunohistochemical detection of p27 and Ki67 expression. Low power (×10) view of clear positive (brown stain) in epithelium of *H. pylori* infected of gastric diffuse adenocarcinoma sample. Primary antibody anti-p27, secondary antibody anti-Ki67 with peroxidase, DAB chromogen and hematoxylin stain

Demonstrations of positive IHC control, and negative and positive staining performed using antibodies against p27kip1 low power (×10), are presented in Figs. 2, 3, 4. Immunohistochemistry indicated that 56/77 (72.7%) of the gastric cancer samples were negative for p27 gene expression. Among the 56 negative samples, 27 were from females and 29 from males. Thirty cases of the negative IHC samples were of diffuse-type gastric cancer, while the remaining 26 were intestinal type. The cellular staining between p27 expression and *H. pylori* genes is shown below.

### PCR Results

All samples were positive for GAPDH, indicating that the test samples contained high-quality DNA and were suitable for further analysis. Regarding the *ureA* gene, 42 out of 77 samples were positive for the *H. pylori ureA* gene; however, no correlations between the *ureA* gene presence and the cancer type or gender were found. The majority of samples (37/42) positive for the *ureA* gene were negative for p27 protein expression (see Table 4), with statistical significance (p<0.05) indicative that the p27 protein was suppressed in almost all gastric cancer samples that were positive for *H. pylori* infection and *ureA* gene. The presence of virulence genes and their combinations are summarized (see Table 4).

**Table 4:** Association between PCR of *ureA* gene and IHC results of p27 expression

*H. pylori* Detection			
P27 expression	Negative ureA gene	Positive ureA gene	Total
IHC negative for p27	19	37	56
IHC positive for p27	16	5	21
Total	35	42	77
Statistical significance	p value > 0.05	p value < 0.05	p value < 0.05

No statistically significant correlations between gene occurrence, gender, or type of gastric cancer were found in this histological analysis; however, all virulence genes demonstrated significance (*p*-value < 0.05) correlation with protein p27 expression based on IHC and PCR data (Table 6).

## Discussion

*Helicobacter pylori* is one of the most common bacterial infections in humans that may progress to gastritis, duodenal ulcers, peptic ulcer disease (PUD), gastric adenocarcinoma, and MALT associated lymphoma [2]. In the current study, we investigated the potential correlation between *H. pylori* infection and gastric adenocarcinoma in a Jordanian population. Although several studies have been conducted in Jordan to investigate the relationship between *H. pylori* infection and gastric cancer [50–52], to the best of our knowledge, this is the first study of its kind in the Middle East to look at the prevalence of *H. pylori* in patients with gastric cancer using gastrectomy samples rather than biopsies. Seventy-seven samples of adenocarcinoma gastrectomy, formalin-fixed, paraffin-embedded tissue samples were collected from the archives of the Pathology Department at JRMS and KAUH (Amman and Irbid cities, respectively) (Table 1). The samples were examined for the presence of past *H. pylori* infection, the prevalence of *cagA* and *vacA* allelic subtypes, and their correlation with one another, as well as with the expression of the tumor suppressor protein, p27.

Our data confirmed the presence of *H*. *pylori* in 42 out of 77 (54.5%) gastrectomy samples, using PCR amplification of the *ureA* gene (see Table 5), while the *cagA* virulence gene was detected in these samples in 57.1% (24/42) of cases. A total of 42 samples positive for the *ureA* gene were further processed for the amplification of *cagA* and *vacA* alleles (s1, s2, m1, m2) (Table 6).

**Table 5:** *H. pylori (ureA* +*) (vacA* +*),* genotypes in gastrectomy tissue samples (p-value > 0.05)

Genotype	Prevalence (%)
*vacAs1* (*n* = 77)	19 (24.7%)
*vacAs2* (*n* = 77)	17 (22.1%)
*vacAm1* (*n* = 77)	11 (14.3%)
*vacAm2* (*n* = 77)	11 (14.3%)
*ureA-vacA*- (*n* = 77)	19 (24.6%)

**Table 6:** Associations of virulence genes with *p27* gene expression (*p* value < 0.05)

Positive virulence gene	P27 expression		*P* value
	Positive	Negative	
*cagA* (*n* = 24)	1	23	< 0.05
*vacAs1* (*n* = 19)	1	18	< 0.05
*vacAs2* (*n* = 17)	3	14	< 0.05
*vacAm1* (*n* = 11)	0	11	< 0.05
*vacAm2* (*n* = 11)	0	11	< 0.05

According to our study, the predominant *vacA* genotypes of patients with *H. pylori* infection in the Jordanian population were s1: 24.7%, s2: 22.1%, m1: 14.3%, and m2 positive: 14.3%. On the other hand, we investigated the link between the expression of p27 and *H. pylori* infection. Our results showed that 56/77 (72.7%) of gastric adenocarcinomas tested negative for p27 expression (see Figure 3 and Table 6). Furthermore, 37 of 42 gastric adenocarcinoma tissue samples that were positive for *ureA* were also negative for p27 expression, with a significant correlation (*p*-value 0.05) (Table 4). Studies showed that *cagA* positivity rates and clinical outcomes differ between countries and population groups [53]. In general, the *cagA* prevalence rate has been found to be 50–60% in Middle Eastern countries [54, 55]. In our study, the *cagA* prevalence rate was 57.1%, whereas 100% of *H. pylori* strains in East Asian countries are *cagA*-positive [56]. From our results, we can demonstrate that virulent *H. pylori* strains associated with adenocarcinoma often carry *cagA*, as more than half of the cases under study were positive for the *H. pylori* strain carrying the *cagA* gene. Our results agree with those of a study performed in Japan, Korea, the United States, and Colombia that reported predominance of the *cagA* genotypes [53–59]. The different combinations of *vacA* s and m regions identify virulence characteristics of the *H. pylori* strain. It has been shown that type s1m1 strains can produce higher cytotoxin activity *in vitro* than type s1m2 strains, whereas the s2m2 strains do not produce detectable amounts of the cytotoxin and thus are considered less virulent [60]. Therefore, it is significant to identify the *vacA* profiles of the isolated strains and then evaluate the subtype combinations, together with the clinical outcome of the patient. According to the results of our study, the predominant genotype was *vacAm1s1*—unlike in other Middle Eastern countries, for example where the predominant subtype was reported to be *vacAm1s2* in Turkey [55]; but similar to the results of other countries like South Africa and Mexico [58, 61]. The study of genotypes in four different countries reported that the *vacAs1ml* genotypes were predominant in Japan, Korea, the United States, and Colombia [54]. The same study reported a higher prevalence of the *vacA* s1 than the *vacA* s2 genotype. A study from Germany showed the most frequent allelic combinations were s1m2: 47.7%, s1m1: 35.4%), and s2m2: 15.4% [60]. Our results showed that 72.7% of gastric adenocarcinoma samples were negative for p27 expression, which means that the lack of p27 expression can be an essential change during gastric carcinogenesis. More recent research indicates differential resistance of anti-microbial therapeutics worthy of further investigation between cagA positive and vacA positive *H. pylori* [62]*.*

Our results confirm a strong correlation between the presence of *H. pylori* in gastric adenocarcinoma and a lack of p27 expression as 37 out of 42 samples with positive *ureA* were negative for p27 expression with a significant association (*p* = 0.001). This result is consistent with a study done in vitro by Shirin et al., who found a strong correlation between the presence of *H. pylori* and the inhibition of p27 immunoexpression [63]. Although prior studies have found a correlation between different carcinoma types and expression of p27, we believe this is the first study to document this using validated viable gastrectomy samples. Therefore, this method of ascertaining individual phenotypes is viable to determine historical *H. pylori* strains. Furthermore, all samples were collected between 2003 and 2013 and validated by PCR and immunohistochemical analysis occurring between 2005 and 2013. Existing protocols at the time of project were indicative that longer PCR primers could potentially be analyzed for up to 20 years and antigenic degradation can occur of immunohistochemical samples similarly [64–67]. Whereas at that time we utilized an additional GAPDH assay to quantify DNA quality. In our combined analysis we show that FFPE could potentially be valid in combination with PCR utilizing FFPE immunohistochemistry on gastrectomy samples between 8 and 10 years. In addition, we utilized a well characterized ki67 protein marker IHC as a standard known marker of cell proliferation.

## Limitations

The limitations are: PCR testing of unknown phenotypes, testing of gastric adenocarcinoma precursor condition tissues to assess p27 expression and other results, stratified or regression analyses by demographics, cancer sub-type, and prior treatment of *H. pylori* with antibiotics.

## Conclusion

Based on these results, we concluded that the suppression of p27 expression and *H. pylori* infection in men and women of all ages were equally correlated and may be associated with the occurrence of gastric adenocarcinomas. Additionally, the expression of the *vacA* genotype does not however, appear to be associated with the inhibition of p27. The absence of p27 expression was found to have a propensity for the *cagA* (+) *H. pylori* genotype, despite the negative correlation. Additional research would help to firmly establish this association and pave the way for new perspectives for diagnosis, prognostic biomarkers, and targeted or individualized therapeutics. As p27 may represent a key diagnostic marker and predictor of adenocarcinoma prognosis in such individuals and may find use for guiding subsequent treatment decisions.

## Supplementary Information

Below is the link to the electronic supplementary material.

Supplementary file1 (DOCX 26 KB)

## Data Availability

Data contained within the article or supplementary materials are also available upon request. Suhaila A. Al-Sheboul ORCiD (0000-0001-9001-3232). Further information on paraffin and formalin embedding; Paraffin Embedding Technique; A Review of Preanalytical Factors Affecting Molecular, Protein, and Morphological Analysis of Formalin-Fixed, Paraffin-Embedded (FFPE) Tissue: How Well Do You Know Your FFPE Specimen? (2014). Archives of Pathology & Laboratory Medicine (allenpress.com); Immunofluorescence Staining of Paraffin Sections Step by Step (2020); Adenosquamous carcinoma; PowerPoint Presentation (esmo.org); cagPAI in UniProtKB search (378)|UniProt; vacA helicobacter pylori in UniProtKB search (3794)|UniProt.

## Abbreviations

*IHC*: Immunohistochemistry
*H*. *pylori*: *Helicobacter* *pylori*
FFPE: Formalin-fixed, paraffin-embedded
*CagPAI*: Cytotoxin-associated pathogenicity island
*VacA*: Vacuolating cytotoxin
*JUST*: Jordan University of Science and Technology
*KAUH*: King Abdullah University Hospital
*JRMS*: Jordanian Royal Medical Services*:*
*GAPDH* Gene: Glyceraldehyde 3-phosphate dehydrogenase gene
