# Supplementary material for: A Genetic and Immunohistochemical Analysis of Helicobacter pylori Phenotypes and p27 Expression in Adenocarcinoma Patients in Jordan
Source: J Epidemiol Glob Health. 2023 Apr 18;13(2):212–25. doi: 10.1007/s44197-023-00099-z (PMC10272050; doi:10.1007/s44197-023-00099-z)
Supplement: Supplementary file 1 — Supplementary file1 (DOCX 26 KB) [file 44197_2023_99_MOESM1_ESM.docx]

***Supplementary Data***

A Genetic and Immunohistochemical Analysis of *H. Pylori* Phenotypes and *p27* expression in Adenocarcinoma Patients in Jordan

Suhaila A. Al-Sheboul ^1,^*, Ahmad Abdul-Razzak Mohammad ^1^, Yasemin Shboul ^1^, Brent Brown ^2^, and Ismail I. Matalka ^3^

Table S1: Results of IHC staining of gastric cancer patients.

|  |  | **Samples** | **Percent** |
| --- | --- | --- | --- |
| **IHC** | **Negative** | 56 | 72.7% |
|  | **Positive** | 21 | 27.3% |
|  | **Total** | 77 | 100% |

**Table S2:** Distribution of p27 positive IHC results among various forms of gastric cancer.

| **Type of Cancer** | **P27 Detection** | | **Total** |
| --- | --- | --- | --- |
|  | **Negative** | **Positive** |  |
| Intestinal | 30 | 7 | 37 |
|  | 81.1% | 18.9% |  |
| Diffused | 26 | 14 | 40 |
|  | 65.0% | 35.0% |  |

**Table S3:** Distribution of the ureA gene sequence in patients with gastric cancer.

|  |  | **Frequency** | **Percent** |
| --- | --- | --- | --- |
| ***ureA* gene** | **Negative** | 35 | 45.5% |
|  | **Positive** | 42 | 54.5% |
|  | **Total** | 77 | 100.0% |

**Table S4:** Correlation between the IHC results and the PCR of the ureA gene sequence.

| **P27 expression** | ***H.pylori* Detection** | | **Total** |
| --- | --- | --- | --- |
|  | **Negative** | **Positive** |  |
| IHC Negative for p27 | 19 | 37 | 56 |
|  | 33.9% | 66.1% |  |
| IHC Positive for p27 | 16 | 5 | 21 |
|  | 76.2% | 23.8% |  |

**Table S5:** Distribution of cagA gene sequence of *H. pylori*-positive gastric cancer patients.

|  |  | **Frequency** | **Percent** |
| --- | --- | --- | --- |
| ***cagA* gene** | **Negative** | 18 | 23.4% |
|  | **Positive** | 24 | 31.2% |
|  | **Total** | 42 | 54.5% |
| [ | |  |  |

**Table S6:** Association between PCR of *cagA* gene sequence and IHC results

| **P27 expression** | ***cagA* detection** | | **Total** |
| --- | --- | --- | --- |
|  | **Negative** | **Positive** |  |
| IHC Negative for p27 | 14 | 23 | 37 |
|  | 37.8% | 62.2% |  |
| IHC Positive for p27 | 4 | 1 | 5 |
|  | 80.0% | 20.0% |  |

**Table S7:** Distribution of the vacAs1 gene sequence in gastric cancer patients with *H. pylori* infection**.**

|  |  | **Frequency** | **Percent** |
| --- | --- | --- | --- |
| ***vacAs1* gene** | **Negative** | 23 | 29.9% |
|  | **Positive** | 19 | 24.7% |
|  | **Total** | 42 | 54.5% |
|  | |  |  |

**Table S8:** Correlation between PCR of *vacA*s1gene sequence and IHC results

| **P27 expression** | ***vacA*s1 Detection** | | **Total** |
| --- | --- | --- | --- |
|  | **Negative** | **Positive** |  |
| IHC Negative for p27 | 19 | 18 | 37 |
|  | 51.4% | 48.6% |  |
| IHC Positive for p27 | 4 | 1 | 5 |
|  | 80.0% | 20.0% |  |

**Table S9:** Distribution of *vacA*m1 gene sequence within gastric cancer patients positive for *H.pylori*

|  |  | **Frequency** | **Percent** |
| --- | --- | --- | --- |
| ***vacA*m1 gene** | **Negative** | 31 | 40.3% |
|  | **Positive** | 11 | 14.3% |
|  | **Total** | 42 | 54.5% |
|  | |  |  |

**Table S10:** Distribution of positive *vacA*m1 gene in different types of gastric cancer.

| **Type of Cancer** | ***vacA*m1 Detection** | | **Total** |
| --- | --- | --- | --- |
|  | **Negative** | **Positive** |  |
| Intestinal | 16 | 4 | 20 |
|  | 80.0% | 20.0% |  |
| Diffused | 15 | 7 | 22 |
|  | 68.2% | 31.8% |  |

**Table S11:** Correlation between PCR of *vacA*m1 gene sequence and IHC results.

| **P27 expression** | ***vacA*m1 Detection** | | **Total** |
| --- | --- | --- | --- |
|  | **Negative** | **Positive** |  |
| IHC Negative for p27 | 26 | 11 | 37 |
|  | 70.3% | 29.7% |  |
| IHC Positive for p27 | 5 | 0 | 5 |
|  | 100.0% | .0% |  |

**Table S12:** Distribution of *vacA*m2 gene sequence within gastric cancer patients positive for *H.pylori*.

|  |  | Frequency | Percent |
| --- | --- | --- | --- |
| ***vacA*m2 gene** | Negative | 31 | 40.3% |
|  | Positive | 11 | 14.3% |
|  | Total | 42 | 54.5% |
|  | |  |  |
